# Periodic Fasting and Acute Cardiac Events in Patients Evaluated for COVID-19: An Observational Prospective Cohort Study

**DOI:** 10.3390/nu16132075

**Published:** 2024-06-28

**Authors:** Benjamin D. Horne, Jeffrey L. Anderson, Francois Haddad, Heidi T. May, Viet T. Le, Stacey Knight, Tami L. Bair, Kirk U. Knowlton

**Affiliations:** 1 Intermountain Medical Center Heart Institute, Salt Lake City, UT 84107, USA; jeffreyl.anderson@imail.org (J.L.A.); heidi.may@imail.org (H.T.M.); viet.le@imail.org (V.T.L.); stacey.knight@imail.org (S.K.); tami.bair@imail.org (T.L.B.); kirk.knowlton@imail.org (K.U.K.); 2 Division of Cardiovascular Medicine, Department of Medicine, Stanford University, Stanford, CA 94305, USA; fhaddad@stanford.edu; 3 Cardiovascular Institute, Stanford University School of Medicine, Stanford, CA 94305, USA; 4 Cardiology Division, Department of Internal Medicine, University of Utah, Salt Lake City, UT 84132, USA; 5 Department of Physician Assistant Studies, Rocky Mountain University of Health Professions, Provo 84606, UT, USA; 6 Genetic Epidemiology Division, Department of Internal Medicine, University of Utah, Salt Lake City, UT 84132, USA

**Keywords:** COVID-19, SARS-CoV-2, periodic fasting, therapeutic fasting, intermittent fasting

## Abstract

Background: Periodic fasting was previously associated with greater longevity and a lower incidence of heart failure (HF) in a pre-pandemic population. In patients with coronavirus disease 2019 (COVID-19), periodic fasting was associated with a lower risk of death or hospitalization. This study evaluated the association between periodic fasting and HF hospitalization and major adverse cardiovascular events (MACEs). Methods: Patients enrolled in the INSPIRE registry from February 2013 to March 2020 provided periodic fasting information and were followed into the pandemic (*n* = 5227). Between March 2020 and February 2023, *N* = 2373 patients were studied, with *n* = 601 COVID-positive patients being the primary study population (2836 had no COVID-19 test; 18 were excluded due to fasting <5 years). A Cox regression was used to evaluate HF admissions, MACEs, and other endpoints through March 2023, adjusting for covariables, including time-varying COVID-19 vaccination. Results: In patients positive for COVID-19, periodic fasting was reported by 180 (30.0% of 601), who periodically fasted over 43.1 ± 19.2 years (min: 7, max: 83). HF hospitalization (*n* = 117, 19.5%) occurred in 13.3% of fasters and 22.1% of non-fasters [adjusted hazard ratio (aHR) = 0.63, CI = 0.40, 0.99; *p* = 0.044]. Most HF admissions were exacerbations, with a prior HF diagnosis in 111 (94.9%) patients hospitalized for HF. Fasting was also associated with a lower MACE risk (aHR = 0.64, CI = 0.43, 0.96; *p* = 0.030). In *n* = 1772 COVID-negative patients (29.7% fasters), fasting was not associated with HF hospitalization (aHR = 0.82, CI = 0.64, 1.05; *p* = 0.12). In COVID-positive and negative patients combined, periodic fasting was associated with lower mortality (aHR = 0.60, CI = 0.39, 0.93; *p* = 0.021). Conclusions: Routine periodic fasting was associated with less HF hospitalization in patients positive for COVID-19.

## 1. Introduction

Intermittent fasting refers to a collection of dietary regimens whose common feature is repeated short-term episodes involving the cessation of energy intake [1]. Common fasting practices include not eating for 16 h per day every day (i.e., time-restricted eating) or not eating 1–3 days per week (e.g., twice-per-week fasting or alternate-day fasting) [2,3,4,5,6,7]. Typically, these regimens are calorie-free during the fasting period [2,3,7], while some modify the fasting with a very low calorie intake (e.g., 500 kcal about 12 h into a 24 h fast) [4,5,6]. Intermittent fasting reduces weight to a similar extent as standard caloric restriction diets and in most cases does so safely [4,5,6], leading to the reductions in cardiometabolic risks that weight loss causes. Fasting also may rebalance circadian health and improve cardiometabolic health without weight loss [2,3,7]. A randomized trial of time-restricted eating versus a control in the context of an isocaloric diet improved insulin sensitivity and β cell responsiveness without weight change, and also decreased blood pressure and oxidative stress [2]. Further, a once-per-week 24 h water-only fast reduced insulin resistance and metabolic syndrome parameters in the fasting arm, while weight change was not different compared to ad libitum non-intervention controls [3].

Periodic fasting is a lower-frequency regimen compared to most intermittent fasting plans. In a series of studies, periodic fasting of about one day per month for religious purposes over an average of 40 years was associated in a pre-pandemic population with greater longevity and a lower incidence of a new heart failure (HF) diagnosis [8]. Fasting may counteract multiple etiologic factors leading to HF [9,10,11], including by lowering blood pressure, reducing cardiac load, improving cardiac contractility, and ameliorating the poor HF prognosis. The HF-risk-modifying impacts of fasting may include altering the human growth hormone/insulin-like growth factor-1 axis [12,13], reducing anemia [12], removing excess sodium and other circulating analytes [8,14,15], activating autophagy [16,17,18,19,20,21], and shifting energy source from glucose to fatty acid-derived ketones [22,23,24,25,26].

Critically, HF is associated with a poor prognosis in people with coronavirus disease 2019 (COVID-19) compared to HF-free individuals [27,28]. An observational cohort study reported that a decades-long history of periodic fasting was associated with a lower risk of hospitalization or mortality after COVID-19 diagnosis [29]. Other data also suggest that fasting may reduce the risk of severe COVID-19 outcomes or the post-acute sequelae of COVID-19 (PASC, or long-COVID) [20,21,30,31]. Whether fasting reduces the risk of severe HF in people with COVID-19 is unknown. This study evaluated the association between routine periodic fasting and hospitalization for the primary diagnosis of HF and with other outcomes in acute and post-acute COVID-19.

## 2. Methods

Objectives and study population: The primary objective of this study was to evaluate whether periodic fasting was associated a with lower risk of HF hospitalization after COVID-19 diagnosis. Other objectives evaluated whether fasting was associated with a lower risk of major adverse cardiovascular events (MACEs), greater longevity, and a lower risk of post-acute outcomes that occurred more than 30 days after COVID-19 diagnosis.

The INSPIRE registry (clinicaltrials.gov, NCT02450006) is a prospective observational research resource that has enrolled coronary angiography patients at Intermountain Health since August 1994. The registry includes electronic research data linked to clinical medical records, longitudinal hospitalization and outpatient outcomes, death records from Intermountain, state, Federal sources, and DNA and plasma samples. Between 7 February 2013, and 16 March 2020, INSPIRE enrolled 5795 patients who reported periodic fasting data and research questionnaire data on socioeconomic, physical activity, alcohol intake, and behavioral parameters. The questionnaire was previously published [8]. Patients underwent angiography for clinical evaluation due to cardiac symptoms or risk factors indicative of potential disease. The INSPIRE registry and this study that used its resources were approved by the Intermountain Institutional Review Board.

**Figure 1:**
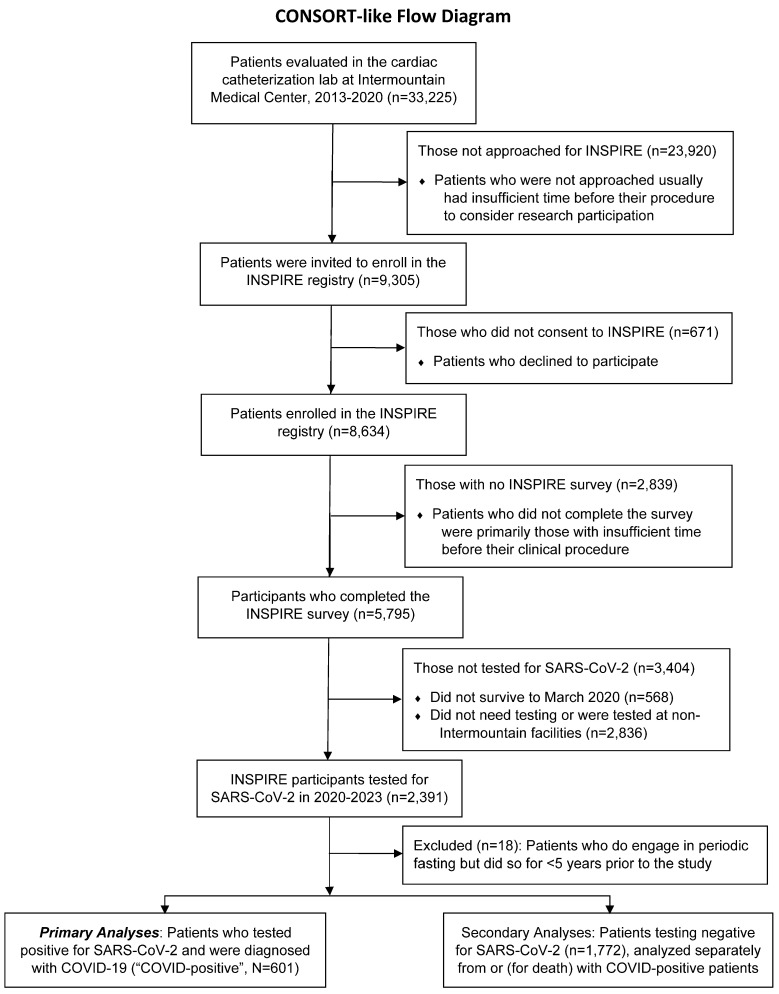
CONSORT-like flow diagram of patients who were included in and excluded from this study.

The first local case of COVID-19 was reported on 6 March 2020; thus, *n* = 568 patients (9.8%) who died prior to that date were excluded. The remaining *n* = 5227 patients were followed into the pandemic to assess their COVID-19 diagnosis, acute events, and PASC. Between 6 March, 2020, and 15 February, 2023, COVID-19 testing was performed in mobile, outpatient, emergency, or inpatient settings for clinical indications, with COVID-19 diagnosed in *n* = 601 (*n* = 1772 were COVID-negative). The *n* = 2836 with no COVID-19 test result in the Intermountain Health electronic medical record were excluded. Figure 1 provides a CONSORT-like diagram of patient exclusions.

Periodic fasting behavior: Periodic fasting behavior was determined in the INSPIRE survey by asking, “Do you routinely abstain from food and drink (i.e., fast) for extended periods of time?” A second question recorded the number of years during life that the patient had engaged in routine fasting [8]. To ensure that the fasting was a long-term behavior, patients had to report a history of 5 years or more of routine periodic fasting; thus, patients with <5 years of fasting (*n* = 18) were excluded. Prior studies showed that about 90% of those reporting periodic fasting fasted for religious purposes and were members of the Church of Jesus Christ of Latter-day Saints (LDS, or Mormon) who fasted for one day about once per month [8]. The other ≈10% fasted for religious purposes using various other fasting frequencies or fasted for health purposes using a variety of more frequent intermittent fasting regimens. Notably, 33–41% of LDS people report periodic fasting [8]; thus, religious affiliation and shared behaviors (e.g., not smoking and alcohol non-use) do not confound fasting associations.

Other covariables: Variables that were collected from the electronic health record at the time of COVID-19 testing for use in study analyses included age, sex, race (self-reported), ethnicity (self-reported), body mass index (BMI), smoking (current or past), hypertension, hyperlipidemia, diabetes, family history of early coronary heart disease, atrial fibrillation, HF history, depression history, anxiety history, prior coronary disease, prior myocardial infarction (MI), cancer history, prior stroke, prior transient ischemic attack, asthma, chronic obstructive pulmonary disease, dementia, peripheral arterial disease, chronic liver disease, renal failure, and prior percutaneous or surgical revascularization. Medication prescriptions at the time of COVID-19 testing were also included. Dates of receipt of a COVID-19 vaccination were drawn from electronic public health databases. From the INSPIRE survey, income, education, marital status, employment status, physical activity, and level of alcohol consumption were included. The presence of coronary artery disease, the location of lesions, and the degree of narrowing were recorded from angiographic findings at the time of the INSPIRE survey.

Study health outcomes: The primary study endpoint was hospitalization for the primary discharge diagnosis of HF on or after the day of COVID-19 diagnosis in the COVID-positive population. This event was registered during the acute and PASC phases of COVID-19 that corresponded to the first 30 days after diagnosis and >30 days after diagnosis, respectively. Event follow-up was conducted through to 14 March 2023. Other longitudinal cardiovascular outcomes in those with COVID-19 included hospitalization for unstable angina, MI, coronary revascularization, stroke, and all-cause mortality. The endpoint of MACEs was defined as a composite of the first outcome of either HF hospitalization or any one of the other cardiovascular outcomes above.

Event data were collected from the Intermountain network of 24 hospitals in Utah and southeastern Idaho. Based on prior unpublished data, >90% of endpoints were captured through Intermountain’s electronic health record, which serves the healthcare needs of more than two-thirds of residents in urban and rural regions across Utah and southeastern Idaho. Further, the minimal endpoints that may be missed due to hospitalization elsewhere should be randomly distributed relative to periodic fasting status. All-cause mortality was identified using electronic health records of Intermountain, Utah death certificates, and the social security death master file, allowing for a complete follow-up of mortal status. Exploratory (i.e., hypothesis-generating) analyses examined the post-acute HF hospitalization, MACEs, and PASC diagnoses that may be sequelae of COVID-19. PASC outcomes were selected based on outcomes that were found in Intermountain electronic data that matched or were similar to the major PASC factors identified by prior work [32].

Statistical approach: The baseline characteristics of the study subjects were evaluated based on periodic fasting status using the chi-square test or Student’s *t*-test, as appropriate. The statistical significance of comparisons between those groups and in analyses of HF hospitalization and other outcomes was at *p*-values of *p* ≤ 0.05, and the analyses were performed in SPSS v.29.0 (IBM SPSS, Inc., Armonk, NY, USA). Analyses other than for the primary endpoint were considered confirmatory of published findings or hypothesis-generating and were not corrected for multiple comparisons.

Raw associations of periodic fasting with HF hospitalization and other longitudinal study outcomes were evaluated graphically using Kaplan–Meier survival curves and the significance of the crude comparison was assessed using the log-rank test. Further analyses utilized Cox regressions to calculate univariable and multivariable adjusted hazard ratios (HRs) and 95% confidence intervals (CIs). Multivariable models for periodic fasting considered the covariables above in stepwise modeling, including a time-varying covariable to model vaccination for COVID-19, since some patients received vaccination prior to the onset of COVID-19 and others received their first dose after their first infection had been resolved. 

Associations of fasting were adjusted in models that entered COVID-19 vaccination first and then added age and other variables in stepwise or forced batch entry up to a maximum of 8–10 variables in a model, given the number of HF hospitalizations. Models entering fasting, COVID-19 vaccination, age, and a single additional covariable were also examined for all of the other study covariables to assess the potential individual confounding effects of each covariable. All factors in Table 1 and Table 2 were considered in building these various multivariable models. For other endpoints, the maximum number of variables included in the model was based on a rule of thumb of 10–15 events per variable. Bivariable models were also evaluated for fasting with other individual covariables to assess each variable’s confounding effect. The final models entered fasting, vaccination, and age, along with statistically significant or confounding covariables. A covariable was defined as a confounder if it induced a change of >10% in the β-coefficient of periodic fasting. Secondary analyses in those testing negative for COVID-19 utilized the Cox regression methods above for HF hospitalization and mortality, but did not consider vaccination status, given the lack of a COVID-19 diagnosis.

## 3. Results

Participant characteristics are provided in Table 1 and Table 2 for individuals (*N* = 601) who were diagnosed with COVID-19. Among the limited number of differences between people who reported periodic fasting and those who reported not fasting, the more significant findings were that fasters were more likely to be non-smokers, not consume alcohol, have a higher education level, have less asthma, and have less COPD. Periodic fasting was reported by 180 (30.0%) of the 601 subjects, and they had fasted routinely for 43.1 ± 19.2 years (min: 7 years, max: 83 years). The time from COVID-19 diagnosis until HF admission or censoring averaged 0.80 ± 0.59 years (maximum: 2.38 years).

**Figure 2:**
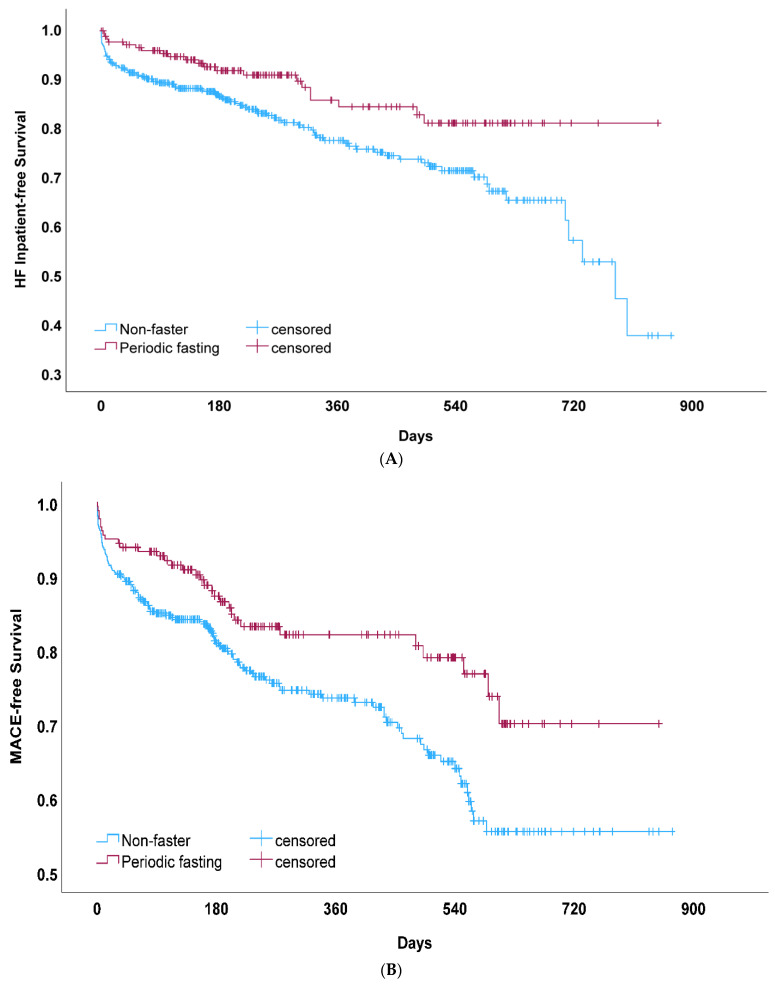
(**A**) Kaplan–Meier survival curves for heart failure (HF) hospitalization among COVID-positive patients who reported participating in periodic fasting compared to those who did not fast (non-fasters). This comparison of crude HF hospitalization-free survival rates measured *p* = 0.014 by the log-rank test. (**B**) Kaplan–Meier survival curves for major adverse cardiovascular events (MACEs) among COVID-positive patients who reported periodic fasting compared to those who did not (non-fasters), which measured *p* = 0.008 by the log-rank test.

HF hospitalization (*n* = 117, 19.5%) was lower (Figure 2A) in fasters compared to non-fasters (13.3% vs. 22.1%; HR = 0.57, CI = 0.37, 0.90; Table 3). The majority of HF hospitalization events were HF exacerbations, with a prior HF diagnosis present for 111 of the 117 HF admits (94.9%). AN adjustment for COVID-19 vaccination had minimal effects on the association between fasting and HF hospitalization (Table 3). Fasting remained protective in multivariable analyses (adjusted HR = 0.63, CI = 0.40, 0.99; *p* = 0.044), including with adjustments for prior HF diagnosis and the other variables in Table 1 and Table 2.

Secondary endpoint analysis for MACEs (Figure 2B) found a lower risk among fasters (17.8% vs. 26.8%; HR = 0.59, Table 3). In multivariable analysis, this association remained significant (adjusted HR = 0.64, CI = 0.43, 0.96). For each MACE endpoint individually (other than HF hospitalization), qualitative differences but a non-significantly lower risk for fasting compared to non-fasters were found for mortality (2.8% vs. 4.0% for fasting vs. non-fasting, respectively; HR = 0.67, CI = 0.25, 1.83; *p* = 0.44), unstable angina (2.2% vs. 3.6%; HR = 0.62, CI = 0.20, 1.86; *p* = 0.39), acute MI (0% vs. 2.1%; HR = 0.028, CI = 0.00, 12.40; *p* = 0.25), revascularization (2.8% vs. 3.8%; HR = 0.56, CI = 0.19, 1.69; *p* = 0.31), and stroke (1.7% vs. 2.4%; HR = 0.67, CI = 0.18, 2.43; *p* = 0.54). Mortality was limited in COVID-positive patients due to a relatively small number of events, but despite this, COVID-19 vaccination was associated with lower mortality (HR = 0.37, CI = 0.15, 0.91; *p* = 0.030), although vaccination was not associated with HF hospitalization (HR = 0.91, CI = 0.61, 1.36, *p* = 0.63) or MACEs (HR = 0.80, CI = 0.56, 1.14; *p* = 0.22). In exploratory analyses of patients with COVID-19, the association between fasting and cardiovascular and PASC outcomes occurring after >30 days post-infection was not statistically significant (Table 4).

**Figure 3:**
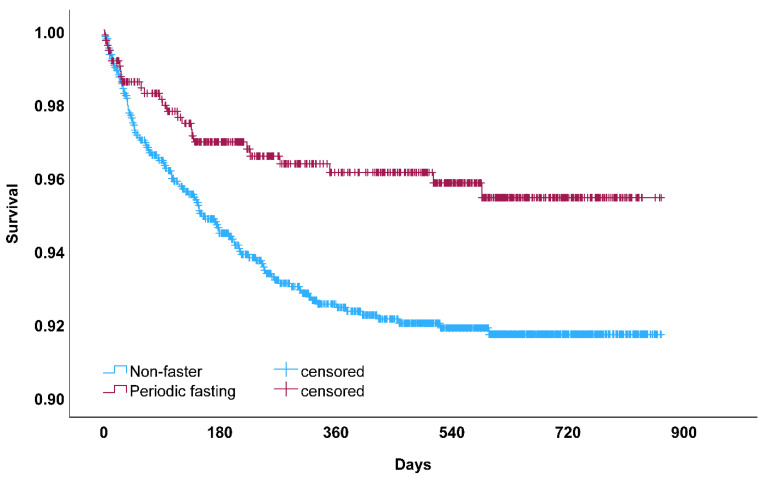
Kaplan–Meier survival curve for all-cause mortality among patients who participated in periodic fasting compared to those who did not (non-fasters) in analyses for all patients testing positive or negative for COVID-19 (*p* = 0.003 by the log-rank test). This association between periodic fasting and greater longevity replicates a pre-COVID finding in another population [8].

Other exploratory analyses examined the *n* = 1771 patients who were COVID-negative (29.7% fasted routinely). These patients were older and more likely to smoke than COVID-positive patients (Supplemental Table S1). In COVID-negative patients, periodic fasting was associated with a lower risk of HF hospitalization [*n* = 360 HF hospitalizations: 15.9% vs. 22.2% for fasting vs. non-fasting; 334 were exacerbations (92.8%); HR = 0.74, CI = 0.58, 0.94; *p* = 0.014]. Fasting was weakly associated with HF hospitalization after adjustment for all covariables except for HF history (adj. HR = 0.79, CI = 0.61, 1.01; *p* = 0.06) and was further reduced when adjusting for HF history (adj. HR = 0.82, CI = 0.64, 1.05; *p* = 0.12). Because overall mortality events were greater when considering patients testing negative for COVID-19 (*n* = 1771; mortality: 3.8% vs. 7.6% for fasting vs. non-fasting, respectively), these were combined with the *n* = 601 COVID-positive (see prior paragraph) for a total of 2372 patients, and periodic fasting was found in this combined patient set (Figure 3) to be associated with greater longevity in both univariable (HR = 0.53, CI = 0.35, 0.82; *p* = 0.004) and multivariable analyses (HR = 0.60, CI = 0.39, 0.93; *p* = 0.021).

## 4. Discussion

Summary: Among patients with a COVID-19 diagnosis, those who reported an average 43-year history of periodic fasting had a 37% lower risk of being hospitalized for the primary diagnosis of HF during an average of 10 months in post-COVID follow-up. Almost all HF hospitalizations (95%) were exacerbations in people with a prior HF diagnosis. Periodic fasting was also associated with a 36% lower risk of MACE in COVID-positive patients, with the majority of events being HF hospitalizations and lesser numbers of each of five other endpoints. In exploratory analyses, fasting was not associated with the heterogeneous PASC endpoints, although many hazard ratios were well below 1.0. For people testing negative for COVID-19, periodic fasting was not associated with HF hospitalization after adjustment for other factors, but asymptomatic patients may not have been tested during active infection and may have had COVID-19 at another time during the study. In the combined population of COVID-positive and COVID-negative patients (*n* = 2373), though, greater longevity was demonstrated, with periodic fasters having a 40% lower risk of mortality, validating pre-pandemic evidence that fasting extends longevity [8].

Context of fasting in heart failure and COVID-19: While HF is one of the comorbid conditions that increases the risk of poor outcomes in people with COVID-19 [27,28], periodic fasting may reduce the incidence and progression of HF [29] and may counteract the effects of COVID-19 [20,21,30,31]. Fasting may modify weight, recalibrate circadian rhythms, and modify metabolism and cardiac function [1,2,3,4,5,6,7]. Such fasting modifications to health may reduce acute COVID-19 outcomes and PASC, including HF exacerbations. The mechanisms of these benefits may include effects on HF that reduce the risk of poor COVID-19 outcomes and effects on COVID-19 that prevent strain on the heart and the unmasking of simmering cardiac dysfunction.

Fasting-related benefits in HF may include various counteractions to HF risk [9,10,11], including metabolic, circulatory, energy management, and functional improvements. Fasting regulates several hormonal and metabolic pathways and may produce benefits to not only the metabolism but also in lowering blood pressure, reducing cardiac load, improving myocyte contractility, and ameliorating organ-level declines in cardiac strength. An established but incompletely understood pathway of HF risk involves the human growth hormone/insulin-like growth factor-1 axis [9]. Evidence is emerging that fasting directly modulates the growth hormone [12,13], with 5- to 14-fold increases in growth hormone during a 24 h fast [12], that may improve cardiac function. Anemia is also a risk factor for HF progression [10], and fasting acutely increases hemoglobin during fasting [12,15]. Further, fasting induces natriuresis—the selective excretion of sodium [12,14,15], and impacts the concentrations of other analytes in the circulation as the body attempts to retain sufficient blood volume [15]. This should reduce B-type natriuretic peptide, as one study reported [8]. Fasting also shifts energy source from glucose to fatty acid-derived ketones, beginning about 12 h into a fast, with substantial ketone elevation after more than 20 h of fasting [26,33]. When ketone exposure is of limited duration and magnitude (keeping levels low to avoid ketoacidosis), as in day-long fasting, it may increase cardiac contractility and limit the deterioration of myocardial strength [11,26,34]. Thus, during fasting and across repeated episodes of routine fasting, multiple key pathways may be improved that limit HF.

Crucially, the metabolic effects of fasting potentially reduce insulin resistance and metabolic syndrome risk, even when weight loss is not different between fasting and control subjects [3]. Improved insulin resistance tracks with reductions in angiotensin-converting enzyme 2 (ACE2), and this may have a direct effect on COVID-19 risk, given the role of ACE2 in binding the severe acute respiratory syndrome coronavirus 2 pathogen [35,36]. The non-fasting group studied here may have already been more frail at the time of COVID-19 diagnosis, permitting them to become more affected by the infection and making their cardiovascular system more susceptible to such a physiological insult. It may further be that anti-inflammatory effects of fasting were in effect already among patients with a history of HF who were fasting routinely, thus preventing HF exacerbation via pre-existing lower levels of inflammation that were not as affected by COVID-19.

Other effects of fasting in counteracting COVID-19 include a variety of aids to the immune system and microbiome [36], and the activation and enhancement of autophagy [17,18,19,20,21]. Autophagy has a multiplicity of potential HF-associated effects but also directly counteracts the effect of COVID-19. For example, the autophagy-regulating transcription factor EB was activated by fasting in an animal model of HF and was shown to reverse cardiomyopathy [16]. And, while COVID-19 deactivates autophagy, fasting enhances autophagic response [20,21]. Because of this, people who fast routinely should experience a more rapid and profound recovery from COVID-19 and have a lower risk of HF exacerbations triggered by COVID-19. Finally, fasting may reduce risk through changes to energy management involving modulated ATP production, greater mitochondrial respiration, increased mitophagy and mitochondriogenesis, and reduced inflammation [22,23,24,25,26,37,38,39].

Strengths and limitations: This observational study of periodic fasting evaluated a non-randomized dietary practice primarily followed for religious purposes, so its observational design may be a limitation. While fasting may cause health benefits, the design of this study does not allow for the determination of causal effects. Of note, that design limitation does not mean the connection is not causal, and indeed the findings are consistent with what randomized controlled trials of intermittent fasting regimens with minimal weight change suggest would occur over the long term [3,7]. Statistical methods adjusting the association of fasting with health outcomes did not reveal another explanatory factor responsible for the connection between fasting and HF or other cardiovascular endpoints, but some confounders may be incompletely controlled or may be unmeasured by this study. Cox regressions revealed that periodic fasting was associated with the study outcomes, despite adjustments for covariables that were statistically significantly associated with fasting, including a lower prevalence in the non-White fasting group, smoking, diabetes, asthma, COPD, diuretics, anticoagulants, anti-diabetic drug and alcohol consumption, and higher education among fasters. Further, periodic fasting retained an association with the study outcomes in Cox regressions, adjusting for covariables with statistical trends with fasting versus non-fasting (i.e., more COVID-19 vaccination, higher education levels, more employment in low-physical-activity jobs, and less ACEi/ARBs in fasting participants). A strength of this study was that more than 60 potential confounding covariables were considered in the survival analyses.

The scope of this study encompassed patients previously referred for cardiac catheterization who were followed as a cohort over multiple years. Some patients died prior to the onset of the pandemic; thus, this population likely reflects a somewhat healthier set of cardiac patients than the standard catheterization laboratory population. Testing positive for COVID-19 depended on having tested at an Intermountain provider; thus, home testing was not reflected in this study. Patients testing negative for COVID-19 at a specific time may have had mild COVID-19 at another time when they were not tested, so caution should be taken in the interpretation of the results for those classified as negative for COVID-19. Whether the findings here generalize to a non-cardiovascular patient population or to populations with different racial or ethnic characteristics is unclear and requires further study.

Although periodic fasting is a religious teaching, and more than 60% of the source population are LDS, based on external population statistics, fewer than half of LDS in prior studies reported participation in periodic fasting [8]. Factors that are more systematically shared by LDS than fasting (e.g., not smoking and not drinking alcohol) are, thus, shared by both those who reported fasting behavior and those who did not fast, and are unlikely to confound the association between fasting and health outcomes. Fitting this conceptual framework, statistical adjustments for smoking, alcohol consumption, and other factors did not have a confounding effect on associations with fasting here; thus, shared religion-associated factors are less of an issue for fasting than may be commonly assumed.

While mortality was lower in COVID-negative patients who fasted, an association between fasting and HF hospitalization in those patients disappeared after multivariable adjustment. For COVID-negative patients, it may be that those hospitalizations reflected the usual progression of severe HF rather than exacerbations triggered by an infection. Given prior evidence for an association of periodic fasting with a lower risk of incident HF in a pre-pandemic setting [8], the complexity of the connection between fasting and HF onset and severity deserves further attention. It is likely that some of those who never tested positive for COVID-19 did so because they did not engage in subsequent testing or were tested outside of the Intermountain system; thus, some may be mis-specified as never having COVID-19, and the results for COVID-negative patients should be interpreted with caution. In contrast, the HF hospitalizations of people who were COVID-positive had the shared feature of acute infection being a possible trigger for HF exacerbation, where fasting may ameliorate the risk of both HF and infectious disease [8,29,36].

## 5. Conclusions

Routine periodic fasting was associated with a lower risk of HF hospitalization among patients with COVID-19 who were followed for up to 3 years after a COVID-19 diagnosis. Fasting was also associated with a lower risk of MACEs (i.e., HF hospitalization, mortality, hospitalization for unstable angina, acute MI, coronary revascularization, and stroke) in COVID-positive patients and with greater longevity overall. The separation of the fasting and non-fasting survival curves for HF hospitalization and for MACEs began early, around 7–10 days after COVID-19 diagnosis. Given the non-randomized nature of the fasting behavior and the observed differences in covariables between non-fasting and fasting groups, caution is warranted in attempting to assess causality from these data. Future investigations in prospective studies of large populations are needed to evaluate the acute benefits of intermittent fasting after COVID-19 diagnosis and the potentially modest PASC benefits.

## Figures and Tables

**Table 1:** Characteristics of patients diagnosed with COVID-19 from the electronic health record.

Characteristic	Overall (*N* = 601)	Non-Fasting (*n* = 421)	Periodic Fasting (*n* = 180)	*p*-Value
Age (years)	64.3 ± 15.1	64.1 ± 15.0	64.9 ± 15.3	0.53
Sex (female)	36.30%	38.20%	31.70%	0.13
Race (non-White *, self-report)	4.50%	5.70%	1.70%	0.029
Ethnicity (Hispanic, self-report)	3.70%	4.30%	2.20%	0.22
COVID-19 vaccination (any)	81.50%	79.80%	85.60%	0.1
Vaccination before infection	52.90%	53.20%	52.20%	0.83
BMI (kg/m^2^), *n* = 583	30.6 ± 7.2	30.7 ± 7.3	30.1 ± 7.0	0.34
Smoking (current or prior)	26.80%	30.90%	17.20%	<0.001
Hypertension	86.70%	87.60%	84.40%	0.29
Hyperlipidemia	84.50%	85.50%	82.20%	0.31
Diabetes	47.10%	49.90%	40.60%	0.036
Family history of early CHD	12.10%	12.80%	10.60%	0.44
Atrial fibrillation	50.40%	52.00%	46.70%	0.23
Heart failure history	60.60%	62.90%	55.00%	0.07
Depression history	43.40%	45.80%	37.80%	0.07
Anxiety history	41.30%	41.80%	40.00%	0.68
Prior coronary disease	77.00%	77.70%	75.60%	0.57
Prior myocardial infarction	21.60%	21.60%	21.70%	0.99
Prior PCI	27.10%	27.30%	26.70%	0.87
Prior CABG	12.80%	14.00%	10.00%	0.18
Cancer history	17.60%	18.10%	16.70%	0.68
Prior stroke	13.50%	12.40%	16.10%	0.22
Prior TIA	13.80%	13.80%	13.90%	0.97
Asthma	29.30%	33.50%	19.40%	<0.001
COPD	19.80%	23.50%	11.10%	<0.001
Dementia	0.30%	0.20%	0.60%	0.54
Peripheral arterial disease	10.80%	10.50%	11.70%	0.66
Chronic liver disease	31.60%	32.80%	28.90%	0.35
Renal failure	2.00%	1.90%	2.20%	0.8
Pre-COVID medication prescription history				
Statins	78.40%	80.00%	74.40%	0.13
Antiarrhythmics	39.40%	40.40%	37.20%	0.47
ACE inhibitors/ARBs	75.20%	77.40%	70.00%	0.053
Beta-blockers	82.50%	84.30%	78.30%	0.08
Calcium channel blockers	66.10%	67.70%	62.20%	0.19
Diuretics	72.90%	75.50%	66.70%	0.025
Aspirin	56.10%	57.20%	53.30%	0.38
Anticoagulants	89.00%	91.00%	84.40%	0.019
Antiplatelets	55.40%	56.80%	52.20%	0.3
Digoxin	1.20%	1.70%	0%	0.11
Anti-diabetic	59.90%	62.50%	53.90%	0.049
Vitamin D supplement	63.40%	63.90%	62.20%	0.7
Antidepressants	53.70%	55.80%	48.90%	0.12
Medication prescriptions at the time of COVID-19 diagnosis				
Statins	53.10%	54.90%	48.90%	0.18
Antiarrhythmics	11.10%	10.90%	11.70%	0.79
ACE inhibitors	19.10%	20.40%	16.10%	0.22
ARBs	21.10%	22.10%	18.90%	0.38
Beta-blockers	42.90%	45.10%	37.80%	0.1
Calcium channel blockers	20.80%	21.90%	18.30%	0.33
Diuretics	40.60%	43.70%	33.30%	0.018
Anti-diabetic	35.30%	38.00%	28.90%	0.032
Azithromycin	4.00%	4.50%	2.80%	0.32
Corticosteroid	36.90%	38.70%	32.80%	0.17
Remdesivir	10.80%	10.90%	10.60%	0.89
Vitamin D supplement	47.80%	49.40%	43.90%	0.22
Antidepressants	28.10%	29.50%	25.00%	0.27

* Combined due to small numbers, non-White races included people self-reporting as American Indian or Alaska Native, Asian, Black or African American, or Native Hawaiian or Other Pacific Islander. ACE: angiotensin-converting enzyme; ARB: angiotensin receptor blocker; CABG: coronary artery bypass grafting; CHD: coronary heart disease; COPD: chronic obstructive pulmonary disease; COVID: coronavirus disease; PCI: percutaneous coronary intervention; TIA: transient ischemic attack.

**Table 2:** Characteristics of patients diagnosed with COVID-19 from patient-reported data collected through the INSPIRE survey.

Characteristic	Overall (*N* = 601)	Non-Fasting (*n* = 421)	Periodic Fasting (*n* = 180)	*p*-Value
Income (US dollars per year)				
<USD 20,000	6.00%	6.40%	5.00%	0.08
USD20,000-USD49,999	14.30%	14.50%	13.90%	
USD50,000-USD99,999	30.10%	28.30%	34.40%	
≥USD100,000	17.30%	15.40%	21.70%	
Declined/unknown	32.30%	35.40%	25.00%	
Education				
Junior high or less	2.30%	2.10%	2.80%	<0.001
High school	20.60%	25.20%	10.00%	
Some college	25.60%	27.80%	20.60%	
Associate’s degree	11.80%	10.50%	15.00%	
Bachelor’s degree	22.10%	20.00%	27.20%	
Master’s degree	9.50%	7.10%	15.00%	
Doctoral/professional	2.50%	2.10%	3.30%	
Declined/unknown	5.50%	5.20%	6.10%	
Marital status				
Married	76.90%	74.30%	82.80%	
Partner	1.20%	1.40%	0.60%	
Widowed	4.30%	4.50%	3.90%	
Single, never married	7.20%	7.60%	6.10%	
Divorced/separated	8.70%	10.00%	5.60%	
Declined/unknown	1.80%	2.10%	1.10%	
Employment status (and job-related type of physical activity)				
Not employed	42.40%	43.70%	39.40%	0.052
Employed, primarily sitting	27.00%	24.70%	32.20%	
Employed, standing/walking	20.10%	19.20%	22.20%	
Employed, definite effort	7.00%	8.80%	2.80%	
Employed, vigorous effort	1.80%	1.70%	2.20%	
Declined/unknown	1.70%	1.90%	1.10%	
Physical activity (aerobic exercise)				
None	70.70%	72.40%	66.70%	0.24
<1 h per week	5.30%	4.50%	7.20%	
1 to <3 h per week	8.50%	7.40%	11.10%	
3 or more hours per week	11.50%	11.20%	12.20%	
Declined/unknown	4.00%	4.50%	2.80%	
Physical activity (cycling)				
None	84.00%	82.90%	86.70%	0.47
<1 h per week	4.00%	4.30%	3.30%	
1 to <3 h per week	3.70%	3.30%	4.40%	
3 or more hours per week	2.70%	2.90%	2.20%	
Declined/unknown	5.70%	6.70%	3.30%	
Physical activity (walking)				
None	7.80%	6.70%	10.60%	0.5
<1 h per week	9.70%	10.00%	8.90%	
1 to <3 h per week	22.00%	21.60%	22.80%	
3 or more hours per week	58.70%	60.10%	55.60%	
Declined/unknown	1.80%	1.70%	2.20%	
Alcohol consumption (drinks)				
None	75.00%	67.50%	92.80%	<0.001
<1 per week	13.00%	17.60%	2.20%	
1–7 per week	8.20%	10.50%	2.80%	
>7 per week	2.30%	2.90%	1.10%	
Declined/unknown	1.50%	1.70%	1.10%	

**Table 3:** In patients positive for COVID-19 (*n* = 601), the associations between periodic fasting and the primary endpoint of heart failure hospitalization and the secondary endpoint of major adverse cardiovascular events. These endpoints include events occurring during both the acute COVID-19 (0–30 days after COVID-19 diagnosis) and post-acute phases (>30 days after COVID-19 diagnosis).

Association of Periodic Fasting	Univariable	Adjusted for COVID-19 Vaccination	Fully Adjusted Multivariable Model
Heart failure hospitalization (hospitalization for the primary discharge diagnosis of heart failure)			
Hazard Ratio (95% CI)	0.57 (0.37, 0.90)	0.57 (0.37, 0.90)	0.63 (0.40, 0.99) *
*p*-value	0.015	0.015	0.044
Major adverse cardiovascular events (all-cause mortality or hospitalization for the primary diagnosis of heart failure, unstable angina, acute myocardial infarction, coronary revascularization, or stroke)			
Hazard Ratio (95% CI)	0.59 (0.40, 0.87)	0.59 (0.40, 0.88)	0.64 (0.43, 0.96) ^†^
*p*-value	0.008	0.009	0.03

* All factors in Table 1 and Table 2 were considered in building multivariable models (see statistical methods), and the final heart failure hospitalization model entered periodic fasting, time-varying COVID-19 vaccination status, age, race, prior heart failure diagnosis, prior myocardial infarction, history of diabetes, and history of atrial fibrillation. ^†^ The final major adverse cardiovascular events model entered periodic fasting, time-varying COVID-19 vaccination status, age, race, prior heart failure diagnosis, prior myocardial infarction, history of diabetes, history of atrial fibrillation, and history of renal failure. CI: confidence interval; COVID: coronavirus disease.

**Table 4:** Exploratory associations of periodic fasting with various cardiovascular outcomes and post-acute sequelae of COVID-19 occurring more than 30 days after the diagnosis of acute COVID-19 (*n* = 601). These results require further evaluation in other populations and larger sample sizes (endpoints with <15 events are noted in the table).

Outcomes	Univariable		Adjusted for COVID-19 Vaccination	
(All >30 Days Post-COVID)	Hazard Ratio (95% CI)	*p*-Value	Hazard Ratio (95% CI)	*p*-Value
Cardiovascular Endpoints (only those >30 days post-COVID)				
HF Hospitalization	0.87 (0.60, 1.26)	0.46	0.87 (0.60, 1.26)	0.47
MACE	0.89 (0.52, 1.26)	0.52	0.89 (0.63, 1.26)	0.53
PASC Endpoints				
Post-COVID Syndrome *	0.90 (0.35, 2.33)	0.83	0.90 (0.35, 2.31)	0.82
Anosmia	Not evaluated individually (*n* = 1 event)			
Ageusia	Not evaluated individually (*n* = 2 events)			
Malaise (*n* = 14 events)	0.64 (0.18, 2.31)	0.5	0.65 (0.18, 2.34)	0.51
Dyspnea	0.74 (0.46, 1.17)	0.2	0.74 (0.46, 1.17)	0.2
Shortness of Breath	0.62 (0.33, 1.14)	0.12	0.60 (0.32, 1.11)	0.1
Fatigue	1.08 (0.67, 1.74)	0.77	1.06 (0.66, 1.72)	0.8
Cognitive Impairment	0.50 (0.17, 1.47)	0.21	0.49 (0.16, 1.45)	0.2
Palpitations	0.88 (0.39, 2.00)	0.77	0.89 (0.39, 2.02)	0.78
Chest Pain	0.89 (0.55, 1.44)	0.63	0.87 (0.54, 1.42)	0.58
Dizziness	0.92 (0.52, 1.65)	0.79	0.88 (0.49, 1.58)	0.67
Vertigo	Not evaluated individually (*n* = 1 event)			
PASC Composite ^†^	0.90 (0.68, 1.18)	0.44	0.89 (0.67, 1.17)	0.41
PASC Composite 2 ^‡^	0.82 (0.61, 1.11)	0.19	0.81 (0.60, 1.10)	0.17
PASC Composite 3 ^§^	0.69 (0.44, 1.07)	0.1	0.68 (0.44, 1.06)	0.09

* International Classification of Diseases, version 10, code U09.9. ^†^ This “PASC Composite” endpoint included post-COVID syndrome, anosmia, ageusia, malaise, dyspnea, shortness of breath, cognitive impairment, palpitations, chest pain, fatigue, dizziness, and vertigo based on outcomes that were available in Intermountain electronic data that matched or were similar to the PASC factors listed in Table 2 in Thaweethai et al. [32]. ^‡^ The “PASC Composite 2” endpoint is the same outcome as in the note above (^†^) for “PASC Composite,” except fatigue was not included. ^§^ The “PASC Composite 3” endpoint includes only dyspnea, shortness of breath, and cognitive impairment and were chosen post hoc based on their independent hazard ratios, *p*-values, and number of events available. AF: atrial fibrillation; COVID: coronavirus disease; HF: heart failure; MACEs: major adverse cardiovascular events; PASC: post-acute sequelae of COVID-19.

## Data Availability

The data underlying this article cannot be shared publicly due to US privacy laws. The data will be shared upon reasonable request to the corresponding author.

## Supplementary Materials

The following supporting information can be downloaded at: https://www.mdpi.com/article/10.3390/nu16132075/s1, Table S1. Selected characteristics of patients who tested negative for COVID-19.

## References

[1] Patikorn C., Roubal K., Veettil S.K., Chandran V., Pham T., Lee Y.Y., Giovannucci E.L., Varady K.A., Chaiyakunapruk N. (2021). Intermittent fasting and obesity-related health outcomes: An umbrella review of meta-analyses of randomized controlled trials. JAMA Netw. Open.

[2] Sutton E.F., Beyl R., Early K.S., Cefalu W.T., Ravussin E., Peterson C.M. (2018). Early time-restricted feeding improves insulin sensitivity, blood pressure, and oxidative stress even without weight loss in men with prediabetes. Cell Metab..

[3] Bartholomew C.L., Muhlestein J.B., May H.T., Le V.T., Galenko O., Garrett K.D., Brunker C., Hopkins R.O., Carlquist J.F., Knowlton K.U. (2021). Randomized controlled trial of once-per-week intermittent fasting for health improvement: The WONDERFUL Trial. Eur. Heart J. Open.

[4] Trepanowski J.F., Kroeger C.M., Barnosky A., Klempel M.C., Bhutani S., Hoddy K.K., Gabel K., Freels S., Rigdon J., Rood J. (2017). Effect of alternate-day fasting on weight loss, weight maintenance, and cardioprotection among metabolically healthy obese adults: A randomized clinical trial. JAMA Intern. Med..

[5] Schübel R., Nattenmüller J., Sookthai D., Nonnenmacher T., Graf M.E., Riedl L., Schlett C.L., von Stackelberg O., Johnson T., Nabers D. (2018). Effects of intermittent and continuous calorie restriction on body weight and metabolism over 50 wk: A randomized controlled trial. Am. J. Clin. Nutr..

[6] Carter S., Clifton P.M., Keogh J.B. (2018). Effect of intermittent compared with continuous energy restricted diet on glycemic control in patients with type 2 diabetes. A randomized noninferiority trial. JAMA Netw. Open.

[7] Jamshed H., Steger F.L., Bryan D.R., Richman J.S., Warriner A.H., Hanick C.J., Martin C.K., Salvy S.J., Peterson C.M. (2022). Effectiveness of early time-restricted eating for weight loss, fat loss, and cardiometabolic health in adults with obesity. JAMA Intern. Med..

[8] Bartholomew C.L., Muhlestein J.B., Anderson J.L., May H.T., Knowlton K.U., Bair T.L., Le V.T., Bailey B.W., Horne B.D. (2021). Association of periodic fasting lifestyles with survival and incident major adverse cardiovascular events in patients undergoing cardiac catheterization. Eur. J. Prev. Cardiol..

[9] Arcopinto M., Bobbio E., Bossone E., Perrone-Filardi P., Napoli R., Sacca L., Cittadini A. (2013). The GH/IGF-1 axis in chronic heart failure. Endocr. Metab. Immune Disord. Drug Targets.

[10] Roger V.L. (2021). Epidemiology of heart failure. A contemporary perspective. Circ. Res..

[11] Wende A.R., Brahma M.K., McGinnis G.R., Young M.E. (2017). Metabolic origins of heart failure. JACC Basic Transl. Sci..

[12] Horne B.D., Muhlestein J.B., Lappé D.L., May H.T., Carlquist J.F., Galenko O., Brunisholz K.D., Anderson J.L. (2013). Randomized cross-over trial of short-term water-only fasting: Metabolic and cardiovascular consequences. Nutr. Metab. Cardiovasc. Dis..

[13] Johnson S.C. (2018). Nutrient sensing, signaling and ageing: The role of IGF-1 and mTOR in ageing and age-related disease. Subcellular Biochemistry.

[14] Spark R.F., Arky R.A., Boulter P.R., Saudek C.D., O’Brian J.T. (1975). Renin, aldosterone and glucagon in the natriuresis of fasting. N. Engl. J. Med..

[15] Kamel K.S., Lin S.H., Cheema-Dhadli S., Marliss E.B., Haperin M.L. (1998). Prolonged total fasting: A feast for the integrative physiologist. Kidney Int..

[16] Ma X., Mani K., Liu H., Kovacs A., Murphy J.T., Foroughi L., French B.A., Weinheimer C.J., Kraja A., Benjamin I.J. (2019). Transcription factor EB activation rescues advanced αB-crystallin mutation-induced cardiomyopathy by normalizing desmin localization. J. Am. Heart Assoc..

[17] Alirezaei M., Kemball C.C., Flynn C.T., Wood M.R., Whitton J.L., Kiosses W.B. (2010). Short-term fasting induces profound neuronal autophagy. Autophagy.

[18] DiNicolantonio J.J., McCarty M. (2019). Autophagy-induced degradation of Notch1, achieved through intermittent fasting, may promote beta cell neogenesis: Implications for reversal of type 2 diabetes. Open Heart.

[19] Abdellatif M., Sedej S., Carmona-Gutierrez D., Madeo F., Kroemer G. (2018). Autophagy in cardiovascular aging. Circ. Res..

[20] Hannan M.A., Rahman M.A., Rahman M.S., Sohag A.A.M., Dash R., Hossain K.S., Farjana M., Uddin M.J. (2020). Intermittent fasting, a possible priming tool for host defense against SARS-CoV-2 infection: Crosstalk among calorie restriction, autophagy and immune response. Immunol. Lett..

[21] Gnoni M., Beas R., Vásquez-Garagatti R. (2021). Is there any role of intermittent fasting in the prevention and improving clinical outcomes of COVID-19?: Intersection between inflammation, mTOR pathway, autophagy and calorie restriction. Virus Dis..

[22] Anton S.D., Moehl K., Donahoo W.T., Marosi K., Lee S.A., Mainous A.G., Leeuwenburgh C., Mattson M.P. (2018). Flipping the metabolic switch: Understanding and applying the health benefits of fasting. Obesity.

[23] Nielsen R., Møller N., Gormsen L.C., Tolbod L.P., Hansson N.H., Sorensen J., Harms H.J., Frøkiær J., Eiskjaer H., Jespersen N.R. (2019). Cardiovascular effects of treatment with the ketone body 3-hydroxybutyrate in chronic heart failure patients. Circulation.

[24] Selvaraj S., Kelly D.P., Margulies K.B. (2020). Implications of altered ketone metabolism and therapeutic ketosis in heart failure. Circulation.

[25] Yurista S.R., Matsuura T.R., Silljé H.H.W., Nijholt K.T., McDaid K.S., Shewale S.V., Leone T.C., Newman J.C., Verdin E., van Veldhuisen D.J. (2021). Ketone ester treatment improves cardiac function and reduces pathologic remodeling in preclinical models of heart failure. Circ. Heart Fail..

[26] Takahara S., Soni S., Maayah Z.H., Ferdaoussi M., Dyck J.R.B. (2022). Ketone therapy for heart failure: Current evidence for clinical use. Cardiovasc. Res..

[27] Yonas E., Alwi I., Pranata R., Huang I., Lim M.A., Gutierrez E.J., Yamin M., Siswanto B.B., Virani S.S. (2020). Effect of heart failure on the outcome of COVID-19—A meta analysis and systematic review. Am. J. Emerg. Med..

[28] Chidambaram V., Tun N.L., Haque W.Z., Majella M.G., Sivakumar R.K., Kumar A., Hsu A.T.W., Ishak I.A., Nur A.A., Ayeh S.K. (2020). Factors associated with disease severity and mortality among patients with COVID-19: A systematic review and meta-analysis. PLoS ONE.

[29] Horne B.D., Muhlestein J.B., May H.T., Le V.T., Bair T.L., Knowlton K.U., Anderson J.L. (2022). Association of periodic fasting with lower severity of COVID-19 outcomes in the SARS-CoV-2 pre-vaccine era: An observational cohort from the INSPIRE registry. BMJ Nutr. Prev. Health.

[30] Grundler F., Mesnage R., Cerrada A., de Toledo F.W. (2023). Improvements during long-term fasting in patients with long COVID—A case series and literature review. Front. Nutr..

[31] Mey J.T., Kirwan J.P., Axelrod C.L. (2023). The role of nutrition in mitigating the effects of COVID-19 from infection through PASC. Nutrients.

[32] Thaweethai T., Jolley S.E., Karlson E.W., Levitan E.B., Levy B., McComsey G.A., McCorkell L., Nadkarni G.N., Parthasarathy S., Singh U. (2023). Development of a definition of postacute sequelae of SARS-CoV-2 infection. JAMA.

[33] Deru L.S., Bikman B.T., Davidson L.E., Tucker L.A., Fellingham G., Bartholomew C.L., Yuan H.L., Bailey B.W. (2021). The effects of exercise on β-hydroxybutyrate concentrations over a 36-h fast: A randomized crossover study. Med. Sci. Sports Exer..

[34] Horton J.L., Davidson M.T., Kurishima C., Vega R.B., Powers J.C., Matsuura T.R., Petucci C., Lewandowski E.D., Crawford P.A., Muoio D.M. (2019). The failing heart utilizes 3-hydroxybutyrate as a metabolic stress defense. JCI Insight.

[35] Cauwenberghs N., Prunicki M., Sabovčik F., Perelman D., Contrepois K., Li X., Snyder M.P., Nadeau K.C., Kuznetsova T., Haddad F. (2021). Temporal changes in soluble angiotensin-converting enzyme 2 associated with metabolic health, body composition, and proteome dynamics during a weight loss diet intervention: A randomized trial with implications for the COVID-19 pandemic. Am. J. Clin. Nutr..

[36] Horne B.D., Bunker T. (2022). Pathogenic mechanisms of the severe acute respiratory syndrome coronavirus 2 and potential direct and indirect counteractions by intermittent fasting. Nutrients.

[37] Walton C.M., Jacobsen S.M., Dallon B.W., Saito E.R., Bennett S.L.H., Davidson L.E., Thomson D.M., Hyldahl R.D., Bikman B.T. (2020). Ketones elicit distinct alterations in adipose mitochondrial bioenergetics. Int. J. Mol. Sci..

[38] Mehrabani S., Bagherniya M., Askari G., Read M.I., Sahebkar A. (2020). The effect of fasting or calorie restriction on mitophagy induction: A literature review. J. Cachexia Sarcopenia Muscle.

[39] Traba J., Geiger S.S., Kwarteng-Siaw M., Han K., Ra O.H., Siegel R.M., Gius D., Sack M.N. (2017). Prolonged fasting suppresses mitochondrial NLRP3 inflammasome assembly and activation via SIRT3-mediated activation of superoxide dismutase 2. J. Biol. Chem..

