# Supplementary material for: Periodic Fasting and Acute Cardiac Events in Patients Evaluated for COVID-19: An Observational Prospective Cohort Study
Source: Nutrients. 2024 Jun 28;16(13):2075. doi: 10.3390/nu16132075 (PMC11243697; doi:10.3390/nu16132075)
Supplement: Supplementary file 1 [file nutrients-16-02075-s001.zip › nutrients-3066354-supplementary.pdf]

Supplemental Table S1. Selected characteristics of patients who tested negative for COVID-19, with comparison between fasting and non-fasting individuals* as well as comparison to the characteristics of those who tested positive for COVID-19 (see Table 1)†.

Characteristic	Overall (N=1,771)	Non-fasting (n=1,244)	Periodic Fasting (n=527)	*p*-value*	*p*-value†
Age (years)	66.7±13.8	66.7±14.0	66.6±13.3	0.88	<0.001
Sex (female)	40.6%	40.3%	41.4%	0.65	0.07
Race (non-White‡, self-report)	5.8%	6.1%	5.1%	0.42	0.56
Ethnicity (Hispanic, self-report)	3.9%	3.9%	4.0%	0.90	0.79
BMI (kg/m^2), n=583	30.8±7.7	30.8±7.6	31.0±7.7	0.50	0.84
Smoking (current or prior)	34.7%	41.1%	19.7%	<0.001	<0.001
Hypertension	86.4%	87.9%	82.9%	0.005	0.93
Hyperlipidemia	82.9%	83.7%	81.02%	0.18	0.52
Diabetes	44.9%	48.3%	36.8%	<0.001	0.40
Family history of early CHD	13.8%	14.0%	13.5%	0.78	0.74
Atrial fibrillation	52.3%	53.4%	49.7%	0.16	0.28
Heart failure history	56.9%	59.6%	50.5%	<0.001	0.15
Depression history	41.7%	44.1%	36.2%	0.002	0.34
Anxiety history	39.4%	42.7%	31.5%	<0.001	0.28
Prior coronary disease	77.1%	78.6%	73.4%	0.018	0.88
Prior myocardial infarction	21.2%	21.9%	19.4%	0.22	0.74
Prior PCI	28.1%	29.3%	25.2%	0.08	0.64
Prior CABG	11.8%	12.9%	9.3%	0.034	0.53
Cancer history	21.4%	22.2%	19.5%	0.22	0.036
Prior stroke	12.3%	12.9%	11.0%	0.28	0.54

Prior TIA	11.9%	11.5%	12.9%	0.40	0.28
Asthma	30.0%	30.4%	29.2%	0.63	0.71
COPD	20.8%	23.6%	14.0%	<0.001	0.51
Dementia	1.3%	1.4%	0.8%	0.30	0.06
Peripheral arterial disease	9.8%	11.3%	6.3%	0.001	0.39
Chronic liver disease	27.3%	28.9%	23.3%	0.016	0.044
Renal failure	1.3%	1.7%	0.4%	0.036	0.038

**p*-value comparing fasting and non-fasting in patients testing negative for COVID-19; †*p*-value comparing between patients testing positive (see data in Table 1) and negative (data in this table) for COVID-19; ‡Combined due to small numbers, non-White races included people self-reporting as American Indian or Alaska Native, Asian, Black or African American, or Native Hawaiian or Other Pacific Islander.

CABG: coronary artery bypass grafting; CHD: coronary heart disease; COPD: chronic obstructive pulmonary disease; COVID: coronavirus disease; PCI: percutaneous coronary intervention; TIA: transient ischemic attack
